# Towards Truly Integrated Undergraduate Radiology Education: An Ultrasound-Based Bedside Radiology Teaching Quality Improvement Project

**DOI:** 10.7759/cureus.96588

**Published:** 2025-11-11

**Authors:** Abdullah Khalil, Tanvirul Islam

**Affiliations:** 1 Medical Education, Bedfordshire Hospital NHS Foundation Trust, Bedfordshire, GBR; 2 Medical Education, Dartford and Gravesham NHS Trust, London, GBR

**Keywords:** experiential learning, radiology curriculum integration, radiology teaching, ultrasound-based learning, undergraduate medical education, undergraduate radiology

## Abstract

Background: Radiology is central to modern clinical practice, yet its integration into undergraduate medical curricula is often limited to lecture-based formats, leading to fragmented learning and low student engagement. To address this gap, we undertook a quality improvement project (QIP) introducing a novel ultrasound-based, small-group teaching intervention during final-year medical students’ surgical rotations, aiming to enhance engagement, diagnostic reasoning, and confidence in radiology.

Methodology: This QIP was conducted at a district general hospital in the United Kingdom. Final-year medical students (*n* = 44) participated in one-hour, small-group ultrasound sessions delivered by radiology registrars during their four-week general surgery rotation. Learning objectives included understanding the strengths and limitations of ultrasound, integrating real-time imaging into diagnostic reasoning, and improving confidence in communicating with radiologists. Evaluation comprised post-session surveys with Likert-scale and free-text responses, as well as blinded performance assessments during a simulated surgical clerking exercise.

Results: Forty students (91%) completed surveys. All students rated the intervention as superior to previous lecture-based radiology teaching, with 36 (90%) describing it as *much better*. The majority strongly agreed that observing live ultrasound enhanced their understanding (86%) and increased confidence in discussing imaging with radiologists (90%). In the clerking exercise, students who attended the intervention scored significantly higher in the diagnostic imaging domain than those who did not (mean 8.5 vs. 6.9, *P *< 0.05). Qualitative feedback highlighted the value of real-time imaging, small-group interaction, and direct engagement with radiology registrars.

Conclusions: Integrating small-group, ultrasound-based radiology teaching into surgical rotations significantly improved student engagement, diagnostic reasoning, and confidence compared to traditional lecture-based formats. This experiential, context-rich approach offers a practical model for embedding radiology education into undergraduate curricula and may foster more meaningful collaboration between future clinicians and radiologists.

## Introduction

Radiology has become indispensable in modern clinical practice, yet its pedagogy in undergraduate education remains poorly understood [1,2]. Undergraduate radiology teaching often suffers from inadequate integration into the core curriculum, resulting in fragmented or supplementary encounters rather than consistent, embedded learning experiences [3].

Traditional undergraduate radiology education typically relies on didactic lectures delivered by radiologists using image-heavy presentations, which can be challenging to integrate into a clinical context [4,5]. At our institution, radiology education for final-year students has traditionally consisted of a lecture series delivered during the medicine rotation, with no structured radiology exposure during other clinical blocks. Attendance at these lectures was consistently very poor, reflecting low student engagement and possibly also de-prioritization of radiology teaching by students in their final year. Clinicians in surgical and medical specialties at our hospital also reported that graduating students often demonstrate limited understanding of radiology’s diagnostic role, impairing their ability to consult effectively with radiologists [6].

Commensurate with radiology’s centrality to clinical practice, there is a need for structured, integrated teaching that situates radiology within core curricular contexts. Building on prior work showing the effectiveness of point-of-care ultrasound for teaching anatomy [7], we designed a novel learning encounter to integrate radiology education into the surgical curriculum. The key features of ultrasound imaging, which make it suited to these objectives, are its portability and, more crucially, its real-time visualization capabilities [8]. When delivered in small-group tutorials at the bedside, ultrasound-based teaching can facilitate active learning through immediate feedback [9]. This model not only enhances student participation but also promotes deeper understanding through the integration of anatomical knowledge and diagnostic reasoning.

The objective of our novel learning intervention was to enhance not only radiology knowledge but also to investigate whether radiology teaching could be integrated with and enhance students’ learning of clinical reasoning in the context of core rotations such as general surgery. This quality improvement project (QIP) aims to assess the impact on student engagement, knowledge acquisition, and perceived clinical relevance. We aim to evaluate whether this pedagogical strategy addresses the limitations of traditional radiology instruction and aligns with current educational best practices.

## Materials and methods

Setting and participants

The QIP was delivered in a district general hospital in the United Kingdom. The sessions were delivered by radiology registrars from the hospital. The students were all final-year medical students during their four-week general surgery rotation. Each student attended one session during the four-week rotation. 

All students who had still not completed their surgical rotation at the time when the intervention was introduced were timetabled to attend a session; hence, there was no randomization or selection for participation.

A total of 50 students were timetabled to attend the sessions. Forty-four students actually attended and participated in the intervention.

The intervention was introduced in addition to the traditional radiology teaching that the students already received during their previous rotations.

Intervention design

One-hour, small-group sessions (5-8 students) were delivered in ultrasound imaging rooms by radiology registrars. Each student attended a single one-hour session within the four-week rotation. Using a live volunteer, students were taken through the ultrasound visualization of abdominal organs, including the gallbladder, bile ducts, pancreas, and kidneys, while discussing the clinical significance of the images and the role of ultrasound in surgical diagnosis, with a focus on its strengths and limitations. For example, students were encouraged not only to know the diagnostic utility of ultrasound in imaging the common bile duct but, from actually observing the imaging in real time, to also understand why the diagnostic information it can provide about the common bile duct is limited in various contexts. Volunteers were all healthy adults from the faculty and staff, and no patient volunteers were involved in this QIP.

The primary learning objectives were to (1) enhance confidence in discussing imaging requests with radiologists and surgical teams (as this is an expected duty of postgraduate house officers), (2) appreciate the strengths and limitations of ultrasound in diagnosing surgical conditions of the abdomen, (3) integrate real-time imaging experiences of abdominal organs with an understanding of the strengths and limitations of ultrasound in diagnosing surgical conditions of the abdomen, and (4) increase opportunities for meaningful student engagement with radiologists.

The sessions were not designed to teach technical ultrasound skills or the mechanics of the modality.

Evaluation

The impact of the intervention was evaluated through mixed methods, including student surveys and clinical feedback from tutors.

Student feedback was obtained using surveys, which were completed by students after attendance at the session. The surveys were created for this QIP. Students were required to answer a combination of questions using scored components, Likert scale (Strongly disagree to Strongly agree), addressing perceived confidence, learning impact, and session comparison with previous radiology teaching they had received in medical school.

The survey also included an optional free-text response option with a prompt asking students to reflect on whether they found anything uniquely beneficial from this learning experience.

Clinical feedback on the performance of students was collected from surgical registrars. This feedback was routinely collected during a separate simulated surgical clerking experience and was not collected primarily for the purpose of evaluating this QIP. These surgical registrars were not directly aware of our learning intervention and were not informed which student groups had participated in the ultrasound-based session, helping to ensure that student evaluation was blinded to participation in the intervention. As part of this surgical clerking experience, students are scored and receive feedback on various separate domains, including a section on selecting diagnostic imaging and discussing the imaging request with a radiologist. During the clerking experience, students were required to select and justify a diagnostic investigation to their supervising surgical colleague and then also call the on-call radiologist to request the scan. The scores from this section of the surgical clerking exercise provide a more objective measure of student performance than the self-reported ratings provided in the post-session survey.

Formal ethics committee approval was not required for this quality improvement project at our institution. No patient or patient data was used in the design or delivery of this QIP.

## Results

A total of 50 students were timetabled to attend the sessions. Forty-four students attended and participated in the intervention. Forty (91%) completed the post-session survey and were ultimately included in the final analysis.

The feedback from students was overwhelmingly positive, with 36 (90%) of respondents rating *Much better *and 10% (*n* = 4) *Better* when asked to directly rate how this learning encounter compares with past radiology teaching that they have experienced in their course (a lecture-based series).

Table 1 shows how students rated their agreement with various statements exploring their experience of the new learning encounter, using a 5-point Likert scale (Strongly disagree to Strongly agree).

**Table 1:** Rates of student agreement with statements exploring their experience of the new learning encounter.

Survey question	Strongly agree (%)	Agree (%)	Neutral (%)	Disagree (%)	Strongly disagree (%)
Observing live ultrasound enhanced my understanding of the strengths and limitations of ultrasound imaging	87.5 (*n *= 35)	12.5 (*n *= 5)	0	0	0
The opportunity to directly visualize abdominal organs was crucial to my learning during this session	87.5 (*n *= 35)	12.5 (*n *= 5)	0	0	0
I feel more confident in discussing imaging requests with radiologists	90 (*n* = 36)	10 (*n* = 4)	0	0	0
I feel more confident in contributing to discussions with senior colleagues in surgical teams when deciding on imaging choices	75 (*n* = 30)	20 (*n* = 8)	5 (*n* = 2)	0	0
The ability to speak directly with a radiology registrar enhanced my learning during the session	92.5 (*n* = 37)	7.5 (*n* = 3)	0	0	0
This session should be integrated into other rotations (e.g., medicine)	100 (*n* = 40)	0	0	0	0

**Figure 1:**
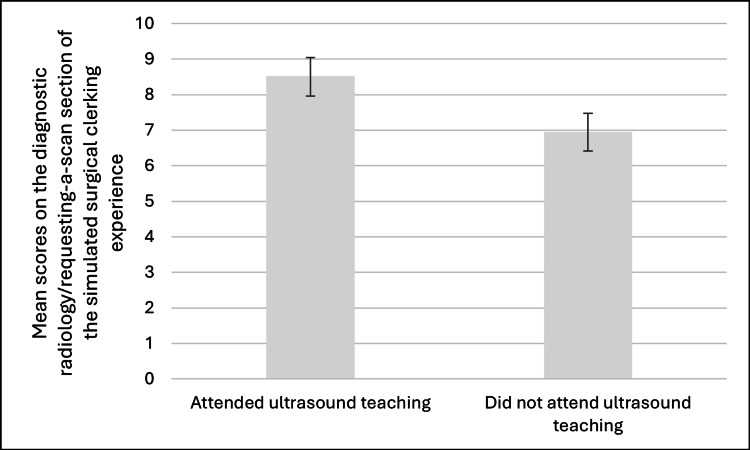
Comparison of mean scores between attendees (n = 24) and non-attendees (n = 31), with a mean difference of 1.56; two-tailed P < 0.05 (calculated using an unpaired t-test). *t*-score = 4.0817.

Clinical feedback scores for performance on the diagnostic radiology section of the separate simulated surgical clerking experience were available for 24 (55%) students who attended the ultrasound-based learning intervention and for 31 students who did not. These scores were reported as a score out of 10. The mean scores for the students who attended the new learning intervention and the students who did not attend are compared in Figure 1.

The mean score of students who attended the ultrasound bedside teaching was significantly higher on the diagnostic radiology/requesting-a-scan domain of the simulated surgical clerking session than students who did not attend (attendees, 8.5 (SD: 1.35) vs. non-attendees, 6.94 (SD: 1.48); mean difference = 1.56; two-tailed *P* < 0.05).

Analysis of the free-text survey responses revealed two key themes regarding the impact of the ultrasound-based radiology teaching: (1) perceptions of radiology as a specialty and (2) active engagement and small-group interaction. 

Perceptions of radiology as a specialty

An unexpected outcome highlighted in the student feedback was the impact on students’ perceptions of radiology as a specialty. Students valued direct interaction with registrars and shared that the experience demystified the specialty, providing insight into the clinical reasoning process of radiologists. Three responses suggested that this was the first occasion in medical school where they engaged critically with a radiologist and that this learning encounter affected their perception of the specialty. One student also specifically commented that this was the first time they had an educational interaction with a Radiology registrar (presumably because traditional undergraduate Radiology teaching is consultant-led at our institution).

Active engagement and small-group interaction

The small-group format was repeatedly cited as a valuable factor in the learning experience. Five students mentioned that this was the first time they experienced radiology teaching in a small group setting. One student expressed that the intimate setting, which enabled meaningful discussions with the radiology registrar, was a crucial part of the learning experience.

## Discussion

Our intervention demonstrates that integrating bedside ultrasound-based radiology teaching into a surgical rotation can significantly enhance final-year medical students’ ability to integrate knowledge of imaging modalities into their diagnostic reasoning, as well as their confidence in discussing investigations with radiologists. Students overwhelmingly rated this intervention as superior to traditional lecture-based radiology teaching, emphasizing the value of direct visualization, real-time feedback, and interaction with radiology registrars. These findings align with previous work highlighting the benefits of experiential, small-group learning in clinical education [3].

The positive impact on students’ confidence in requesting and discussing imaging suggests that situating radiology education within a clinical context can bridge the gap between theoretical knowledge and practical application [10]. By observing live ultrasound imaging, students were able to connect anatomical knowledge with diagnostic reasoning, providing a more integrated learning experience [11]. This supports the concept that active, context-rich teaching methods can improve not only knowledge acquisition but also students’ readiness for postgraduate responsibilities [1,12].

The observed improvement in clinical performance on the simulated surgical clerking exercise provides an objective corroboration of the subjective survey results. Students who attended the ultrasound sessions scored significantly higher on the diagnostic imaging component, suggesting that even a single focused session can have measurable educational benefits. This supports the idea that short, targeted interventions can produce meaningful improvements in clinical competency when delivered in an experiential, contextually relevant format [13].

While these results are promising, several limitations must be considered. First, this QIP was conducted at a single institution with a relatively small cohort, which may limit generalizability to other medical education contexts.

All students who had still not completed their surgical rotation at the time this intervention was introduced were included in the QIP and timetabled to attend the session. Hence, student participation was not randomized for this QIP, but the order in which students are assigned to complete their rotations by the medical school administration is random; hence, we do not expect this to introduce significant bias.

Third, attendance data were not available for prior lecture-based radiology teaching, which students are offered on previous medical rotations, making it difficult to directly compare baseline engagement or prior exposure between groups. Additionally, long-term retention of knowledge and the impact on actual clinical practice post-graduation were not assessed.

Delivering small-group, ultrasound-based teaching within a clinical rotation presents several logistical challenges. Organizing sessions required coordination of space, scheduling, and equipment availability. Importantly, the sessions were delivered by radiology registrars rather than consultants, largely due to the limited availability of senior staff. Interestingly, this reliance on registrars revealed an underutilized teaching resource: many radiology registrars were eager for opportunities to teach and engage with students, yet traditional undergraduate radiology education at our institution is heavily consultant-led [14]. Leveraging registrars not only allowed the sessions to be delivered feasibly within the constraints of the rotation but also fostered a more approachable, interactive learning environment, facilitating meaningful discussion and real-time feedback, which may be more limited in traditional consultant-led sessions [15]. Moreover, the dynamic learning environment meant the radiology registrars were teaching diagnostic reasoning and not only imaging [16].

Despite these limitations and challenges, our findings suggest several practical implications for medical education. Embedding radiology teaching within clinical rotations and using real-time imaging modalities can enhance student engagement, diagnostic reasoning, and confidence with interdisciplinary communication. Future research should explore scaling this intervention across multiple specialties and institutions, assessing longitudinal outcomes, and evaluating cost-effectiveness. Additionally, integrating similar experiential learning opportunities in other imaging modalities, such as CT, is an avenue that should be explored.

## Conclusions

This QIP demonstrates that integrating small-group, bedside ultrasound-based teaching within a surgical rotation can effectively address the limitations of traditional, lecture-based radiology education. By situating radiology in a clinical context and leveraging engaged radiology registrars as facilitators, the intervention enhanced student engagement, clinical reasoning, and confidence in interacting with radiologists. These findings suggest that experiential, context-rich teaching can overcome the fragmented and passive nature of conventional undergraduate radiology instruction, providing a practical model for more integrated and meaningful radiology education in medical curricula.
